# Author Correction: Cell-type specific profiling of histone post-translational modifications in the adult mouse striatum

**DOI:** 10.1038/s41467-023-36768-7

**Published:** 2023-02-22

**Authors:** Marco D. Carpenter, Delaney K. Fischer, Shuo Zhang, Allison M. Bond, Kyle S. Czarnecki, Morgan T. Woolf, Hongjun Song, Elizabeth A. Heller

**Affiliations:** 1 Department of Systems Pharmacology and Translational Therapeutics, University of Pennsylvania, Philadelphia, PA USA; 2 Institute for Translational Medicine and Therapeutics, University of Pennsylvania, Philadelphia, PA USA; 3 Penn Epigenetics Institute, Perelman School of Medicine, University of Pennsylvania, Philadelphia, PA USA; 4 Department of Neuroscience, University of Pennsylvania, Philadelphia, PA USA

**Keywords:** Neuroscience, Epigenetics in the nervous system, Epigenetics

Correction to: *Nature Communications* 10.1038/s41467-022-35384-1, published online 13 December 2022

The original version of this article contained an error in the “Data availability” Statement. The hyperlink provided for the accession number GSE193673 was incorrectly given as https://www.ncbi.nlm.nih.gov/geo/query/acc.cgi?acc=GSE196673 instead of https://www.ncbi.nlm.nih.gov/geo/query/acc.cgi?acc=GSE193673. This has been corrected in the PDF and HTML version of the article.

