# Supplementary material for: Cell-type specific profiling of histone post-translational modifications in the adult mouse striatum
Source: Nat Commun. 2022 Dec 13;13:7720. doi: 10.1038/s41467-022-35384-1 (PMC9747932; doi:10.1038/s41467-022-35384-1)
Supplement: Supplementary file 1 — Supplementary Information [file 41467_2022_35384_MOESM1_ESM.pdf]

Supplementary Figure 1

A A2a and D1 INTACT mouse fitness

	Avg male weight (g)	Avg female weight (g)	Avg. # of litters born	Days to mate when first paired	Avg. # of pups/litter
C57Bl/6J	21.34	17.52	2.89	26.40	5.24
Sun1-GFP; D1-cre	18.23	17.49	3.40	27.80	6.00
Sun1-GFP; A2a-cre	20.33	16.61	3.50	27.67	5.44

B Striatal INTACT mRNA Bioanalyzer

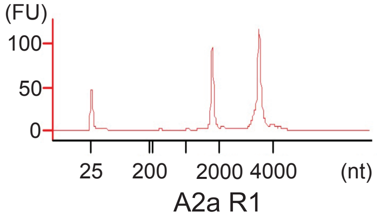

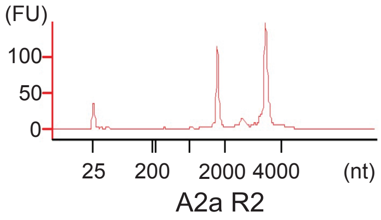

Supplementary Figure 1. INTACT mouse model and RNA extraction following INTACT validation. A) Animal weight and fitness data from Sun1-GFP; D1-cre and Sun1-GFP; A2a-cre mouse lines (P56-57, n = 6-8/group) **B)** Representative mRNA BioA outputs following A2a-INTACT using Agilent Pico RNA kit. RIN 9.80 and 9.70, respectively

Supplementary Figure 2

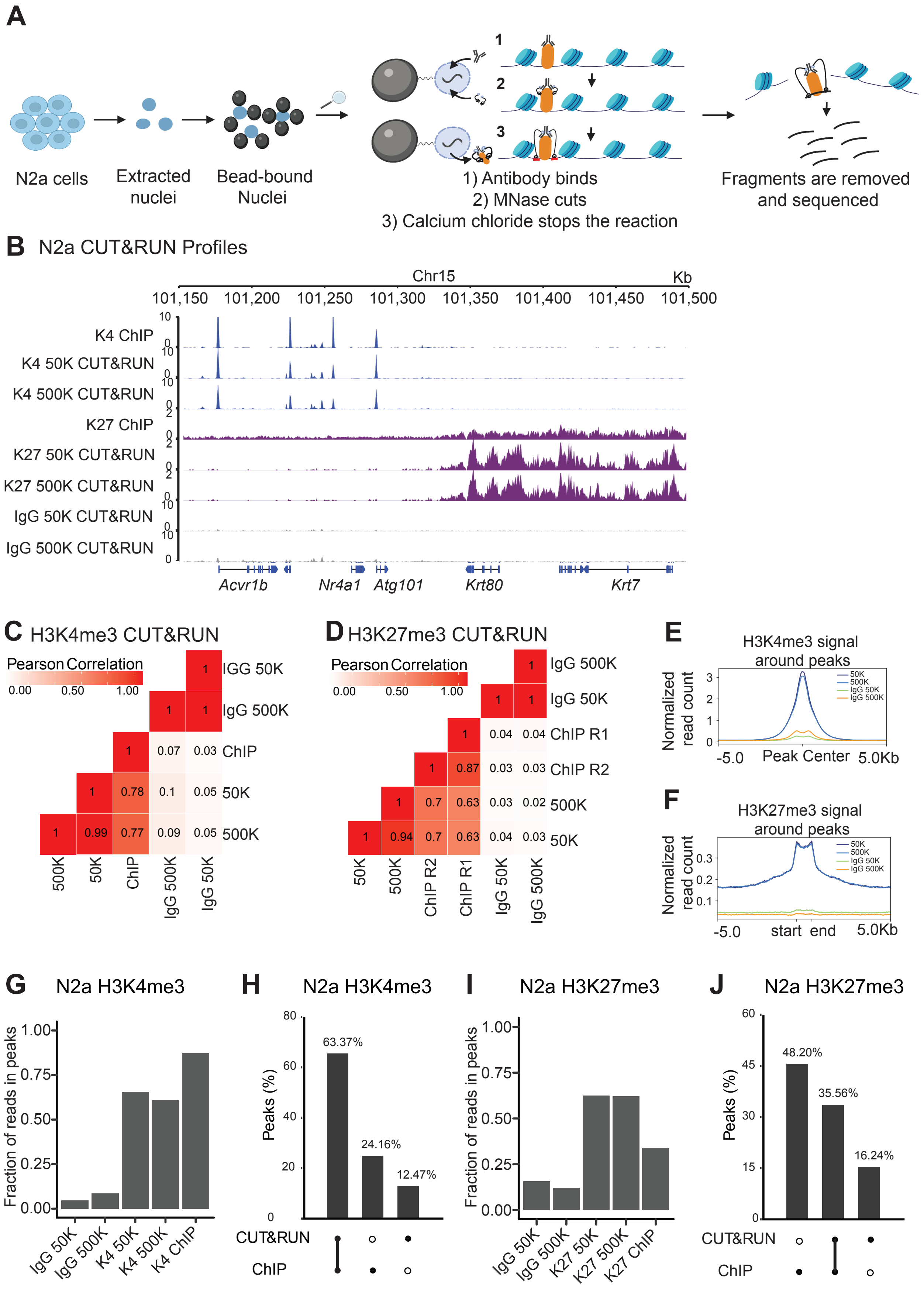

Supplementary Figure 2

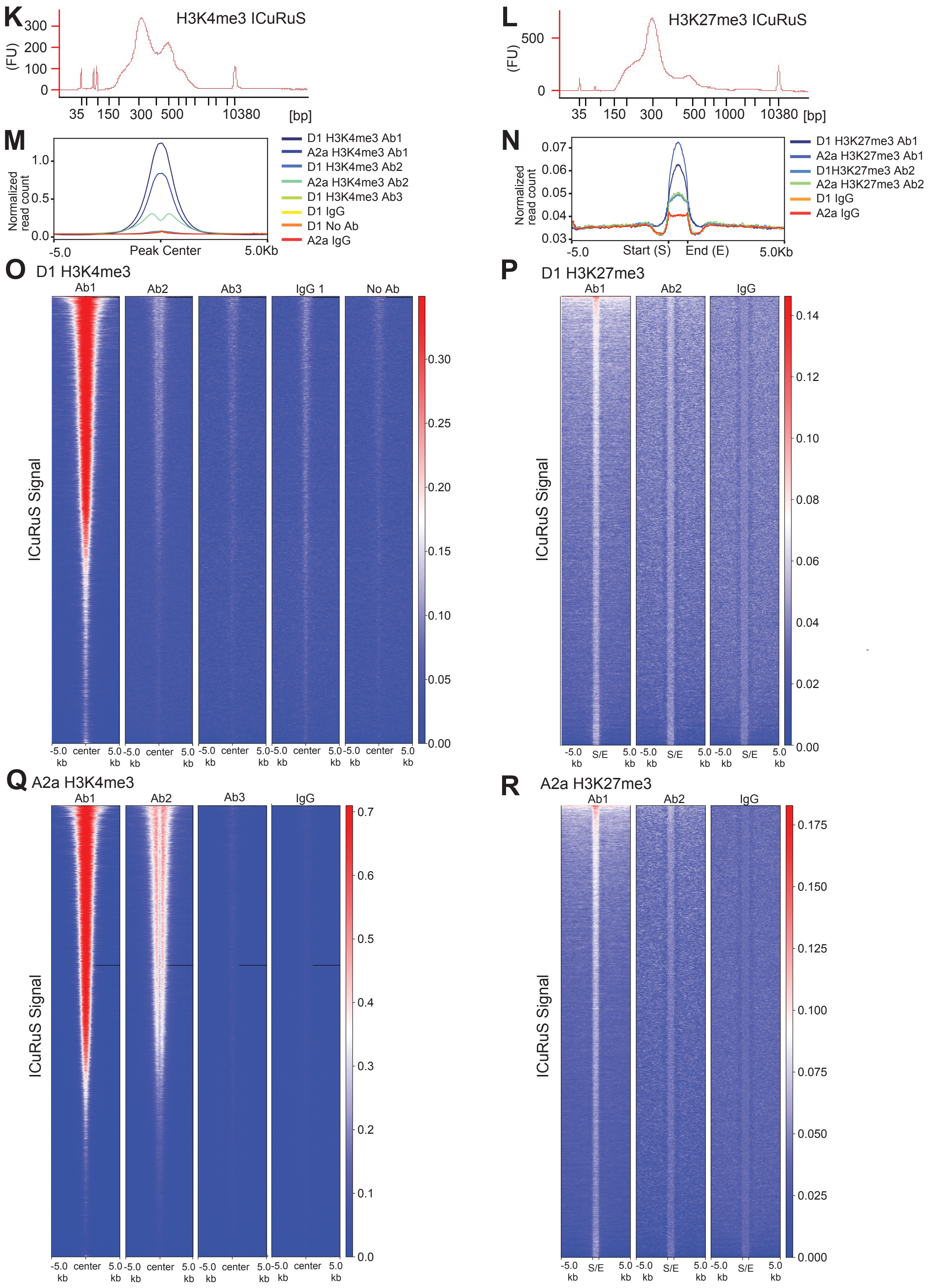

Supplementary Figure 2. CUT&RUN Protocol Validation. A) Schematic of CnR protocol on transfected N2a cells created at Biorender.com. **B)** Representative genome browser views of different profiling methods in N2a cells. Two N2a cell amounts 50,000 (50K) and 500,000 (500K) were tested for the CnR approach. Signal is normalized to total mapped reads: Count Per Million (CPM). N2a ChIP-seq data come from [55] (H3K4me3) and [54] (H3K27me3). **C)** H3K4me3 and **D)** H3K27me3 heatmap showing Pearson's correlation coefficients among N2a ChIP-seq and CnR H3K4me3 signals, and N2a CnR IgG control. The correlation coefficients were calculated by dividing the genome into 2 kb bins and counting reads in each bin. **E)** Averaged N2a H3K4me3 or IgG signal centered on peaks called from N2a H3K4me3 ChIP-seq data by MACS2. **F)** Averaged N2a H3K27me3 or IgG signal around peaks called from N2a H3K27me3 ChIP-seq data by SICER. Peaks within 5kb were merged to avoid double counting. **G)** Fraction of reads in peaks called from N2a H3K4me3 ChIP-seq data for each indicated dataset. **H)** Overlapped and individual peaks between N2a ChIP-seq and CnR H3K4me3 data sets. **I)** Fraction of reads in peaks called from N2a H3K27me3 ChIP-seq data for each indicated dataset. **J)** Overlapped and individual peaks between N2a ChIP-seq and CnR H3K27me3 data sets. **K-L)** Representative Agilent HS DNA kit BioA outputs following library prep for H3K4me3 and H3K27me3 CnR, respectively **M-N)** Density plots of different **(M)** H3K4me3 and **(N)** H3K27me3 antibodies used for A2a- and D1-specific ICuRuS. H3K4me3 Antibody 1 (Ab1): Abcam; H3K4me3 Antibody 2 (Ab2): Active Motif; H3K4me3 Antibody 3 (Ab3): Epicyphr; H3K27me3 Antibody 1 (Ab1): Active Motif; H3K27me3 Antibody 2 (Ab2): Thermo Fisher. **O-P)** Heatmaps from different **(O)** H3K4me3 and **(P)** H3K27me3 antibodies D1-specific ICuRuS. **Q-R)** Heatmaps from different **(Q)** H3K4me3 and **(R)** H3K27me3 antibodies A2a-specific ICuRuS.

Supplementary Figure 3

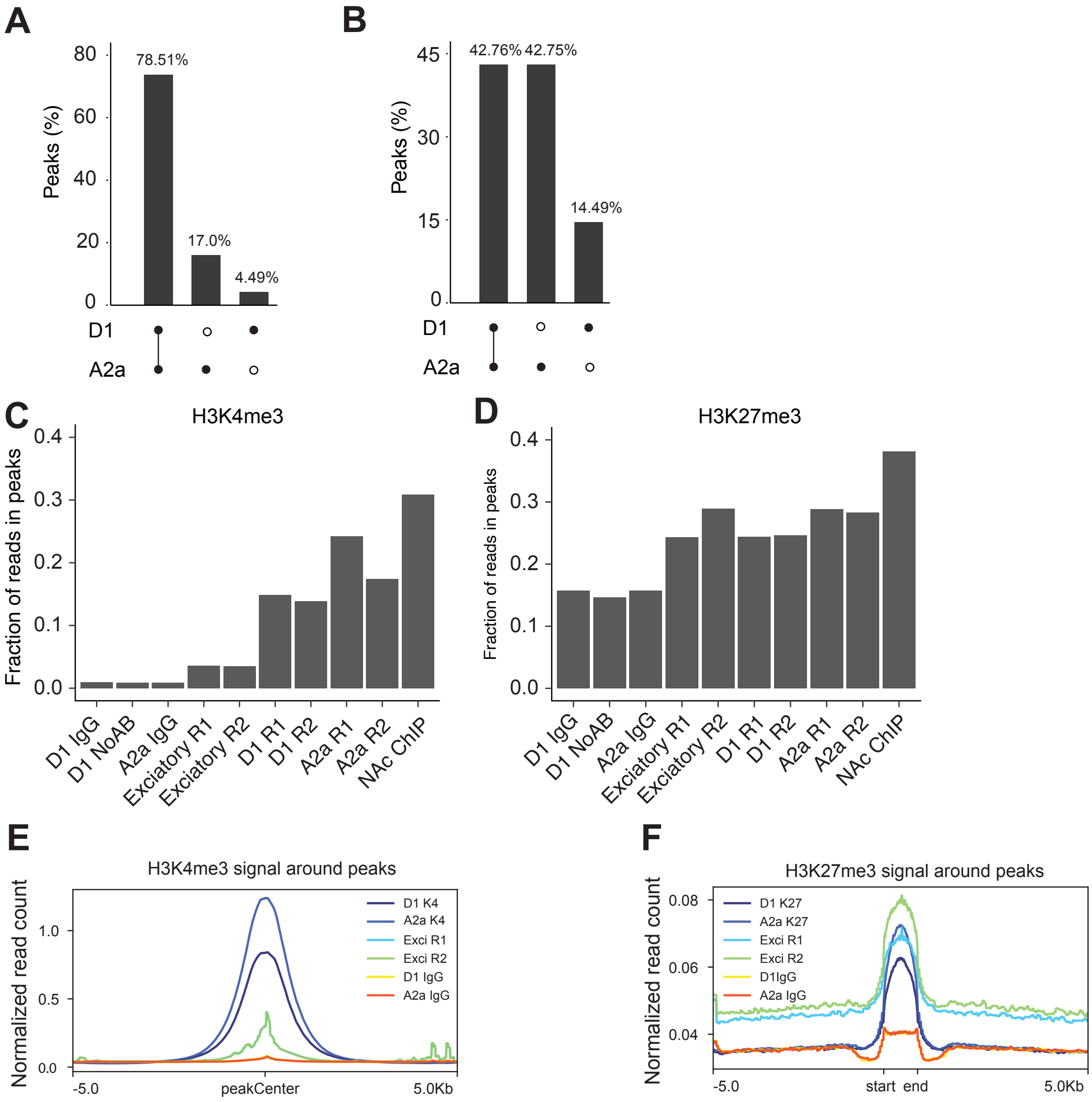

Supplementary Figure 3. ICuRuS epigenomic profiling show expected peak profiles in A2a and D1 nuclei. **A-B)** Overlapped and individualized peaks between A2a and D1 **(A)** H3K4me3 and **(B)** H3K27me3 ICuRuS. **C-D)** Fraction of reads in peaks called from NAc **(C)** H3K4me3 and **(D)** H3K27me3 ChIP-seq data for each indicated dataset. Nucleus accumbens (NAc) bulk ChIP-seq data come from [56]. Excitatory neuron native ChIP-Seq data from [21]. Density plots comparing H3K4me3 (**E**) and H3K27me3 (**F**) signal to native ChIP-Seq from [21].

Supplementary Figure 4

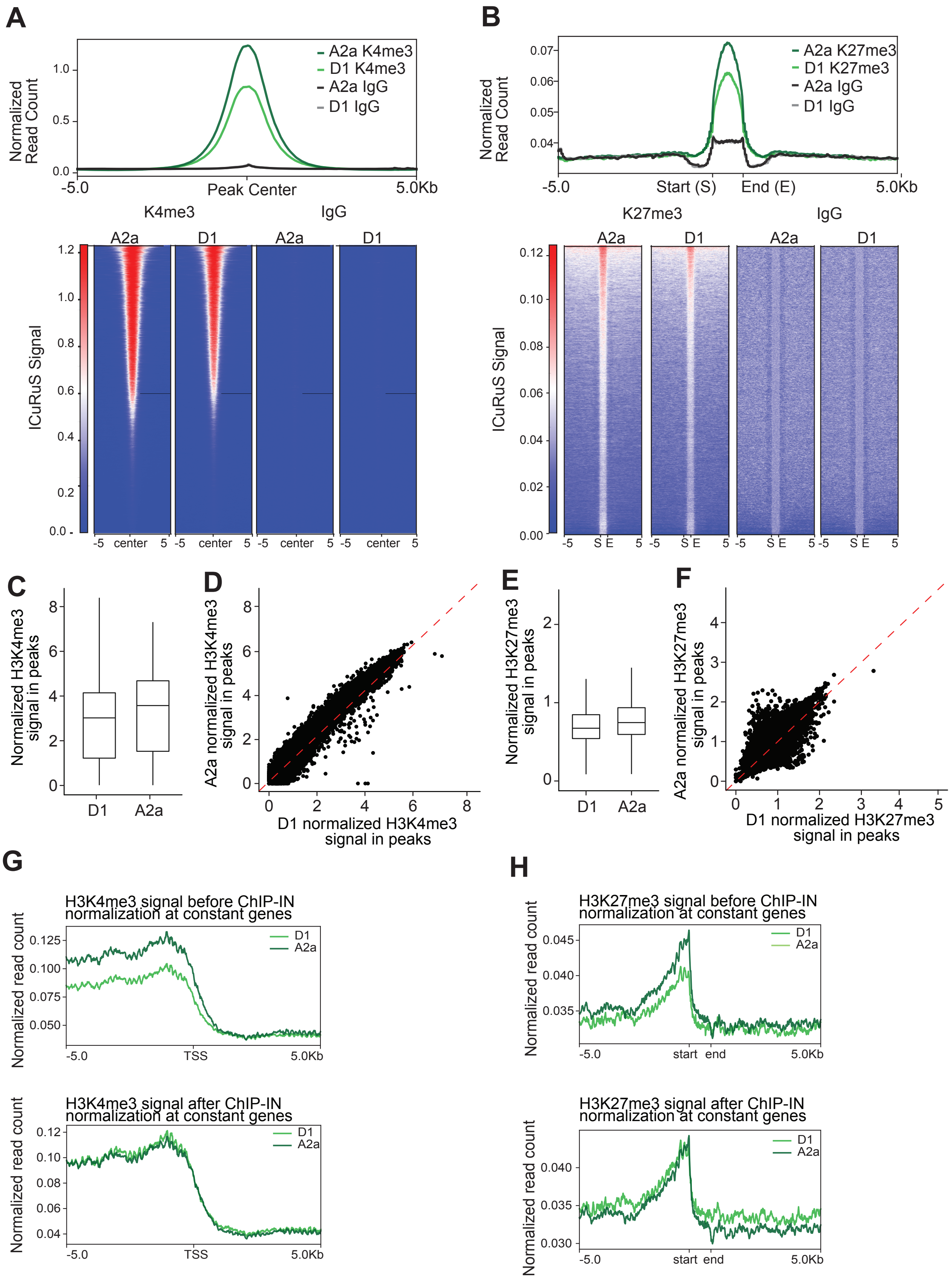

Supplementary Figure 4. ICuRuS epigenomic profiling shows prominent, selective signal in A2a and D1 nuclei. A) A2a and D1 CnR H3K4me3 or IgG signal centered on peaks called from NAc H3K4me3 ChIP-seq by MACS2. Top 20,000 peaks sorted by MACS2 score were used. Heatmap shows signal around individual peaks, and the averaged signal is shown above the heatmap. Two replicates of CnR data were merged. Signal is normalized CPM. **B)** Enrichment of A2a and D1 CnR H3K27me3 or IgG signal around peaks called from NAc H3K27me3 ChIP-seq by SICER. Signal is normalized CPM. Boxplot **(C)** and scatterplot **(D)** showing H3K4me3 signal in peaks called from NAc H3K4me3 ChIP-seq data in A2a and D1 ICuRuS. Signal is normalized to total mapped reads CPM. The boxplot is defined by the mean (middle line), interquartile range (lower and upper quartile represent observations outside the 24 –76 percentile range), and unfilled cycles indicating outliers. Boxplot **(E)** and scatterplot **(F)** showing H3K27me3 signal in peaks called from NAc H3K27me3 ChIP-seq data in A2a and D1 ICuRuS. Signal is normalized to total mapped reads CPM. The boxplot is defined by the mean (middle line), interquartile range (lower and upper quartile represent observations outside the 24 –76 percentile range), and unfilled cycles indicating outliers. **G)** A2a and D1 CnR H3K4me3 signal before and after CHIP-IN normalization, centered on the TSS of transcriptionally constant genes. Signal is normalized by variance across transcriptionally constant genes (quantile normalization). **H)** A2a and D1 CnR H3K27me3 signal before and after CHIP-IN normalization, centered on the TSS of transcriptionally constant genes. Signal is normalized by variance across transcriptionally constant genes (quantile normalization).

Supplementary Figure 5

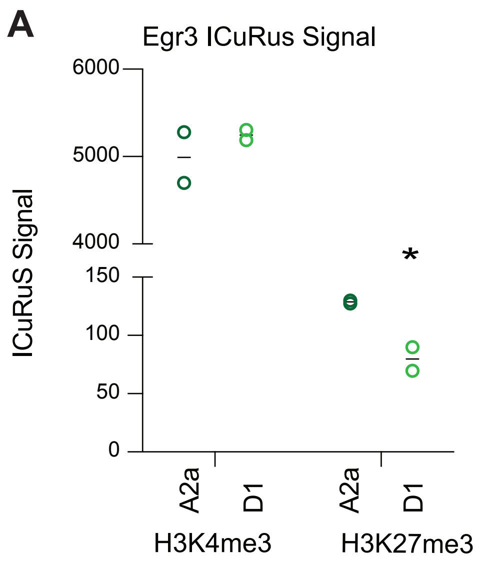

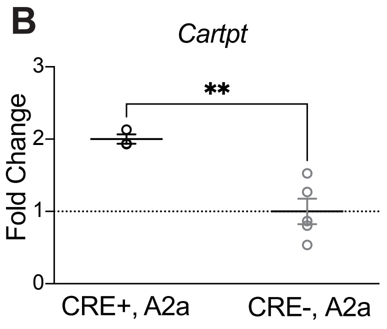

Supplementary Figure 5. ICuRuS signal reveals cell-type specific signal at select genes. A) H3K4me3 and H3K27me3 signal quantified 2 kb upstream and 1 kb downstream of the *Egr3* TSS in both A2a and D1 nuclei (Two-sided ttest H3K4me3: $t_{(4)} = 0.878$, p = 0.476 H3K27me3: $t_{(4)} = 4.794$, p = 0.041; n = 2/group). Adjustment for multiple comparisons were not made. $^{**}P < 0.01$**.** Signal is normalized by variance across transcriptionally constant genes (quantile normalization). **B)** qPCR following A2a-INTACT for A2a-INTACT Cre+ vs A2a-INTACT Cre- (bulk supernatant) for *Cartpt* revealed expression is increased in A2a+ nuclei compared to bulk supernatant ($t_{(6)} = 4.183$, p = 0.0058; n = 2, 5, respectively). $^{**}P < 0.01$.

Supplementary File 1. A2a and D1 ICuRuS Peaks.

Protocol for ICuRuS in mouse brain
All product numbers are in the methods section unless otherwise noted

STEP 1: INTACT [11]

1. Sacrifice animal and collect region(s) of interest using ice-cold Homogenization Buffer (Buffer HB) in the tissue culture dish. Store tissue in 1.5mL tubes, slow freeze on dry ice, and store at -80°C until processing.
2. Add 1.2mL Buffer HB + supplement to the 1.5mL tube containing tissue. Then, transfer tissue and buffer into a 2mL glass dounce homogenizer on ice.
3. Homogenize tissue with the loose pestle (10 pulses).
4. Add 100µL 5% IGEPAL CA-630, and further homogenize with tight pestle (seven pulses).
5. Add 1.3mL of 50% iodixanol density medium to homogenate and resuspend using a P1000 pipette at least five times to thoroughly mix.
6. Pour sample mixture into an empty 13.2 mL, Open-Top Thinwall Ultra-Clear Tube (14 x 89mm) that has been sitting on ice.
7. Underlay the sample mixture with 2.5mL 30% iodixanol solution.
8. Underlay the sample mixture with 1.33mL 40% Iodixanol solution.
9. Seal tubes with parafilm.
10. Spin samples at 13,000 rpm x 18 min at 4°C in in a swinging bucket centrifuge using a SW 41 Ti Swinging-Bucket Rotor (Beckman Coulter, 331362).
11. While the samples are spinning, wash 80µL of Protein G Dynabeads per sample two times with Wash Buffer. For wash buffer volume use up to 5x total bead amount. Resuspend beads in 80µL of Wash Buffer.
12. Pipette 20µL of the 80µL /sample of beads into new Eppendorf tube for pre-clearing. Place the remaining 60µL /sample of washed beads, on an end-to-end rotator at 4°C until use.
13. When spin is finished, vacuum aspirate or remove the 25% iodixanol layer and about 2/3 of the 30% iodixanol layer with a pipette.
14. Collect 1.5mL of the sample band at the 30%-40% iodixanol interface.
15. Place each sample into a labeled pre-clear tube and rotate tubes at 4°C x 15 min. All INTACT steps from this point forward were performed in a 4°C cold room.
16. While each sample is pre-clearing, prepare three tubes for each sample: in two tubes, add 5µL of anti-GFP antibody and 200µL of wash buffer in each tube. The last tube is for final product collection and can be set aside.
17. Once pre-clear is complete, place each pre-cleared sample on magnet for at least five minutes.
18. Add approx. 750µL of each sample (no beads) into each 200µL antibody+wash buffer tube. End-to-end rotate at 4°C x 30 min.
19. Add the remaining 60µL of washed beads/sample to each tube containing antibody+wash buffer+sample (30µL of beads/each tube). End-to-end rotate at 4°C x 20 min.
20. Place samples on/off magnet quickly, adding and removing the sample every 30 seconds for seven cycles. When samples were off magnet, they were completely resuspended by inversion to resuspend beads.
21. Following intervals, place the samples on the magnet for at least two minutes to ensure all beads, which contain the GFP+ sample, are connected to the magnet.
22. Remove and discard the supernatant.
23. Add 800µL wash buffer and remove samples from magnet. Place back on an end-to-end rotator for at least one minute and then place back on magnet. Remove supernatant. After initial wash conduct two more washes.
24. After completing three washes, transfer both tubes of each sample to a single 1.5mL tube on the magnet. Remove and discard the supernatant.

25. Resuspend each sample in 400uL of Wash Buffer and proceed to CUT&RUN.

STEP 2: CUT&RUN [15]

1. Place the samples on the magnet and discard supernatant.
2. Add 100µL of wash buffer, resuspend by pipetting up and down 10 times with a P200 pipet, and transfer to 0.2mL tubes.
3. Place the samples back on the magnet and repeat wash and resuspension.
4. Place the samples on the magnet and discard supernatant.
5. Resuspend the bead-bound nuclei in 50ul digitonin buffer mixed with antibody (1:100) and end-to-end rotate samples for two hours at 4ºC. To keep beads in solution, slightly elevate cap side of the 0.2mL tubes before placing on rotator.
6. Place samples on magnet and discard supernatant.
7. Wash samples 2X with 250µL digitonin buffer (once on and once off the magnet).
8. Add 0.5 µL CUTANA pAG-MNase (20x stock) in 50ul digitonin buffer to each sample and resuspend.
9. Antibody-enriched fragments were cut by a one-hour incubation at 4ºC.
10. Place the samples on the magnet and discard supernatant.
11. Wash samples 2X with 250µL digitonin buffer (once on and once off the magnet).
12. 50 µL of digitonin buffer containing 100mM $CaCl_2$ was added to each sample and the antibody-enriched fragments were released by a two-hour incubation at 4ºC on an end-to-end rotator.
13. Add 33µL Stop Buffer to each sample, and gently vortex to mix.
14. Incubate samples for 10 min at 37ºC.
15. To each sample, add 1 µl of 10% (wt/vol) SDS and 1.5 µl of proteinase K (20 mg/ml). Mix by inversion and incubate for 20 min at 50 °C.
16. Mix each sample with 100 µL phenol-chloroform-isoamyl and transfer mixture to a MaXtract High Density tube (Qiagen, 129056).
17. Centrifuge at 16,000g x five minutes at room temperature.
18. Add 100 µL chloroform sample and invert 10X to mix.
19. Centrifuge at 16,000g x five minutes at room temperature.
20. Remove top liquid phase by pipetting it into to a fresh tube containing 2µL glycogen (2mg/mL).
21. Add 500µL of ice cold 100% ethanol and store at -20 for ≥12 hours.
22. Centrifuge at 16,000g x 15 minutes at 4ºC.
23. Remove the liquid from the tube (do not disturb the pellet at the bottom).
24. Rinse the pellet in 1mL 100% ethanol.
25. Centrifuge at 16,000g x one minutes at 4ºC.
26. Remove ethanol and air dry the pellet for five minutes.
27. Dissolve the pellet in 1:10 diluted TE.

STEP 3: Library Prep [80]

1. Prior to starting the protocol, place AMPure XP beads at room temperature. (They need to be at room temperature for ≥ 30 minutes before use.
2. Add the following components into a sterile, 0.2mL nuclease free tube for each sample: 25 µl CUT&RUN DNA, 1.5 µl NEBNext Ultra II End Prep Enzyme Mix, 3.5 µl NEBNext Ultra II End Prep Reaction Buffer.
3. Resuspend by pipetting up and down ≥ 10 times with a P100 or P200 pipet, and spin down the samples.

4. Place in a thermocycler, with the heated lid set to ≥ 60°C and run the following program: 20°C for 30 minutes, 50°C for 60 minutes, and hold at 4°C until sample removal.
5. 0 minutes, Place the samples on the magnet and discard supernatant.
6. Dilute the Adaptor 1:30 based on the amount you need to use (it will be 1.25 µl/sample).
7. Prep a master mix of 15 µl NEBNext Ultra II Ligation Master Mix and 0.5 µl NEBNext Ligation Enhancer per sample.
8. Add 15.5 µl of the master mix prepped in step 6 and 1.25 µl of the diluted adapter to each sample. (Note: do not add the diluted adaptor the master mix without the sample. Add each component individually.)
9. Resuspend by pipetting up and down ≥ 10 times with a P100 or P200 pipet, and spin down the samples.
10. Place in a thermocycler and incubate at 20°C for 15 minutes.
11. Add 1.5 µl of NEB USER enzyme to the ligation mixture.
12. Resuspend by pipetting up and down ≥ 10 times with a P100 or P200 pipet, and spin down the samples.
13. Place in a thermocycler, with the heated lid set to ≥ 47°C and incubate at 37°C for 15 minutes.
14. Vortex the room temperature AMPure XP beads to resuspend them. (Note: beads must be at room temperature for maximized efficiency).
15. Add 54 µl (~1.1x) AMPure XP beads to each sample following the ligation reaction.
16. Resuspend by pipetting up and down ≥ 10 times with a P100 or P200 pipet, and spin down the samples.
17. Place the samples on a magnet for ≥ five minutes and once solution is clear, carefully remove and discard the supernatant. (Note: do not discard beads.)
18. Add 200 µl of 80% freshly prepared ethanol to each sample while still on the magnet.
19. Incubate at room temperature for ≥ 30 seconds (but not more than two minutes).
20. Carefully remove and discard the supernatant.
21. Repeat step 18 and add 200 µl of 80% freshly prepared ethanol to each sample while still on the magnet.
22. Incubate at room temperature for ≥ 30 seconds (but not more than two minutes).
23. Carefully remove and discard the supernatant. Remove any trace of ethanol with a P10 pipette while on the magnet.
24. Air the dry beads for five minutes with all samples on magnet with the tube lids open. (Note: Set a timer for the five-minute drying session. Do not over dry the beads.)
25. Remove the samples from the magnet and elute the DNA from the beads iby adding 0.1X TE.
26. Mix well by vortex.
27. Incubate for ≥ two minutes at room temperature.
28. Place the samples back on the magnet for ≥ five minutes and once solution is clear, carefully remove and the supernatant and transfer the supernatant from each sample to a new sterile, 0.2mL nuclease free tube.
29. Add the following components to each sample individually: 13 µl Adaptor Ligated Fragments, 15 µl NEBNext Ultra II Q5 Master Mix, 1 µl Universal PCR Primer/i5 Primer, 1 µl Index Primer/i7 Primer (a different ID for each sample).
30. Place in a thermocycler and run the following program: 98°C for 30 seconds, for 13 cycles: 98°C for 10 seconds followed by 65°C for 10 seconds, then separately 65°C for five minutes. Finally, hold at 4°C until sample removal.
31. Once again, vortex the room temperature AMPure XP beads to resuspend them. (Note: beads must be at room temperature for maximized efficiency).
32. Add 33 µl (~1.1x) AMPure XP beads to each sample following the PCR reaction.

33. Resuspend by pipetting up and down ≥ 10 times with a P100 or P200 pipet, and spin down the samples.
34. Place the samples on a magnet for ≥ five minutes and once solution is clear, carefully remove and discard the supernatant. (Note: do not discard beads.)
35. Add 200 µl of 80% freshly prepared ethanol to each sample while still on the magnet.
36. Incubate at room temperature for ≥ 30 seconds (but not more than two minutes).
37. Carefully remove and discard the supernatant.
38. Repeat step 18 and add 200 µl of 80% freshly prepared ethanol to each sample while still on the magnet.
39. Incubate at room temperature for ≥ 30 seconds (but not more than two minutes).
40. Carefully remove and discard the supernatant. Remove any trace of ethanol with a P10 pipette while on the magnet.
41. Air the dry beads for five minutes with all samples on magnet with the tube lids open. (Note: Set a timer for the five-minute drying session. Do not over dry the beads.)
42. Remove the samples from the magnet and elute the DNA from the beads iby adding 0.1X TE.
43. Mix well by vortex.
44. Incubate for ≥ two minutes at room temperature.
45. Place the samples back on the magnet for ≥ five minutes and once solution is clear, carefully remove and the supernatant and transfer the supernatant from each sample to a new sterile, 0.2mL nuclease free tube.
46. Optional: Conduct Agilent BioA for quality control validation of a successful library prep.
47. Store samples at -20°C until sequencing.
