# Supplementary material for: Cell-type specific profiling of histone post-translational modifications in the adult mouse striatum
Source: Nat Commun. 2022 Dec 13;13:7720. doi: 10.1038/s41467-022-35384-1 (PMC9747932; doi:10.1038/s41467-022-35384-1)
Supplement: Supplementary file 3 — Reporting Summary [file 41467_2022_35384_MOESM3_ESM.pdf]

nature portfolio

Corresponding author(s): Elizabth A. Heller

Last updated by author(s): Nov 17, 2022

Reporting Summary

Nature Portfolio wishes to improve the reproducibility of the work that we publish. This form provides structure for consistency and transparency in reporting. For further information on Nature Portfolio policies, see our Editorial Policies and the Editorial Policy Checklist.

Statistics

For all statistical analyses, confirm that the following items are present in the figure legend, table legend, main text, or Methods section.

n/a	Confirmed	
☐	☒	The exact sample size (*n*) for each experimental group/condition, given as a discrete number and unit of measurement
☐	☒	A statement on whether measurements were taken from distinct samples or whether the same sample was measured repeatedly
☐	☒	The statistical test(s) used AND whether they are one- or two-sided<br>*Only common tests should be described solely by name; describe more complex techniques in the Methods section.*
☒	☐	A description of all covariates tested
☒	☐	A description of any assumptions or corrections, such as tests of normality and adjustment for multiple comparisons
☐	☒	A full description of the statistical parameters including central tendency (e.g. means) or other basic estimates (e.g. regression coefficient) AND variation (e.g. standard deviation) or associated estimates of uncertainty (e.g. confidence intervals)
☐	☒	For null hypothesis testing, the test statistic (e.g. *F*, *t*, *r*) with confidence intervals, effect sizes, degrees of freedom and *P* value noted<br>*Give P values as exact values whenever suitable.*
☒	☐	For Bayesian analysis, information on the choice of priors and Markov chain Monte Carlo settings
☒	☐	For hierarchical and complex designs, identification of the appropriate level for tests and full reporting of outcomes
☐	☒	Estimates of effect sizes (e.g. Cohen's *d*, Pearson's *r*), indicating how they were calculated

Our web collection on statistics for biologists contains articles on many of the points above.

Software and code

Policy information about availability of computer code

Data collection	Illumina bcl2fastq2
Data analysis	Bowtie2 v2.1.0, STAR v2.7.1a, cufflinks v2.2.0, deepTools v3.5.0, HOMER v4.11, MACS2 v2.2.7.1, Samtools v0.1.19, R v4.2.2, Picard v2.23.4, SICER v2.0 The custom scripts for the analysis can be found at https://github.com/HellerLAbeats/lCuRuS

For manuscripts utilizing custom algorithms or software that are central to the research but not yet described in published literature, software must be made available to editors and reviewers. We strongly encourage code deposition in a community repository (e.g. GitHub). See the Nature Portfolio guidelines for submitting code & software for further information.

Data

Policy information about availability of data

All manuscripts must include a data availability statement. This statement should provide the following information, where applicable:

- Accession codes, unique identifiers, or web links for publicly available datasets
- A description of any restrictions on data availability
- For clinical datasets or third party data, please ensure that the statement adheres to our policy

All raw and processed sequencing data generated in this study have been deposited to NCBI Gene Expression Omnibus under accession number: GSE193673 (Gene Expression Omnibus under accession number: GSE193673 (https://www.ncbi.nlm.nih.gov/geo/query/acc.cgi?acc=GSE196673). The qPCR and cell counting data generated in this study are provided in the Supplementary Information/Source Data file. The N2a H3K4me3 ChIP-seq: GSE91043, (https://www.ncbi.nlm.nih.gov/geo/query/acc.cgi?acc=GSE91043); N2a H3K27me3 ChIP-seq: GSE107310 (https://www.ncbi.nlm.nih.gov/geo/query/acc.cgi?acc=GSE107310); NAc H3K4me3 and

H3K27me3 ChIP-seq: GSE42811 (https://www.ncbi.nlm.nih.gov/geo/query/acc.cgi?acc=GSE42811), NAc RNA-seq: GSE121199 (https://www.ncbi.nlm.nih.gov/geo/query/acc.cgi?acc=GSE121199). Source data are provided with this paper.

Human research participants

Policy information about studies involving human research participants and Sex and Gender in Research.

Reporting on sex and gender	*Use the terms sex (biological attribute) and gender (shaped by social and cultural circumstances) carefully in order to avoid confusing both terms. Indicate if findings apply to only one sex or gender; describe whether sex and gender were considered in study design whether sex and/or gender was determined based on self-reporting or assigned and methods used. Provide in the source data disaggregated sex and gender data where this information has been collected, and consent has been obtained for sharing of individual-level data; provide overall numbers in this Reporting Summary. Please state if this information has not been collected. Report sex- and gender-based analyses where performed, justify reasons for lack of sex- and gender-based analysis.*
Population characteristics	*Describe the covariate-relevant population characteristics of the human research participants (e.g. age, genotypic information, past and current diagnosis and treatment categories). If you filled out the behavioural & social sciences study design questions and have nothing to add here, write "See above."*
Recruitment	*Describe how participants were recruited. Outline any potential self-selection bias or other biases that may be present and how these are likely to impact results.*
Ethics oversight	*Identify the organization(s) that approved the study protocol.*

Note that full information on the approval of the study protocol must also be provided in the manuscript.

Field-specific reporting

Please select the one below that is the best fit for your research. If you are not sure, read the appropriate sections before making your selection.

[x] Life sciences [ ] Behavioural & social sciences [ ] Ecological, evolutionary & environmental sciences

For a reference copy of the document with all sections, see nature.com/documents/nr-reporting-summary-flat.pdf

Life sciences study design

All studies must disclose on these points even when the disclosure is negative.

Sample size	Sample size was based on prior published data from similar experiments 11, and the high reproducibility between replicates Male and Female mice were allocated equally into groups and sex was not considered in the study design. No sex analysis were performed because the low sample size limits meaningful conclusions.
Data exclusions	No data was excluded from this study.
Replication	2 technical replicates were performed for each assay and were successful.
Randomization	Allocation was random.
Blinding	Investigators were not blinded to the genotype of the mouse because each cell type was processed separately. However, following nuclei isolation samples were de-identified and annotated afterwards.

Reporting for specific materials, systems and methods

We require information from authors about some types of materials, experimental systems and methods used in many studies. Here, indicate whether each material, system or method listed is relevant to your study. If you are not sure if a list item applies to your research, read the appropriate section before selecting a response.

Materials & experimental systems

n/a	Involved in the study
☐	☒ Antibodies
☐	☒ Eukaryotic cell lines
☒	☐ Palaeontology and archaeology
☐	☒ Animals and other organisms
☒	☐ Clinical data
☒	☐ Dual use research of concern

Methods

n/a	Involved in the study
☐	☒ ChIP-seq
☒	☐ Flow cytometry
☒	☐ MRI-based neuroimaging

Antibodies

Antibodies used	IHC: rabbit anti-Drdl (1:500, Bioss USA BSM-52920R) or rabbit anti-A2a (1:200, Fisher Scientific PAl-042), co-incubated with goat anti-GFP (1:500, Rockland, 600-101-215). Sections were then washed three times with PBS and incubated at room temperature in the dark for two hours with secondary antibodies for fluorescent labeling: Donkey anti-Rabbit lgG (H+L) Highly Cross-Adsorbed Secondary Antibody, Alexa Fluor Plus 555 (1:1000, Fisher Scientific A32794) and Donkey anti-Goat lgG (H+L) Cross-Adsorbed Secondary Antibody, Alexa Fluor 488 (1:1000, Fisher Scientific A-11055).<br><br>CnR: anti-H3K4me3 (Antibody 1: 1:50 dilution, Abcam, Ab8580 (Figure 2); Antibody 2: 1:50 dilution, Active Motif, 39159 (Supplemental Figure 2); Antibody 3: 1:50 dilution, Epicyphr, 13-0041), anti-H3K27me3 (Antibody 1: 1:100 dilution, Active Motif, 39055; Antibody 2: 1:100 dilution, Thermo Fisher, MA5-1198) or IgG (1:100; Epicypher, 13-0042)
Validation	Antibodies were previously validated in CnR (Zhu et al. 2021; Skene et al. 2017)

Eukaryotic cell lines

Policy information about cell lines and Sex and Gender in Research

Cell line source(s)	N2a cells from ATCC.
Authentication	Cells were not authenticated.
Mycoplasma contamination	Cells were not tested for mycoplasma.
Commonly misidentified lines (See ICLAC register)	These cells lines were not used in this study

Animals and other research organisms

Policy information about studies involving animals; ARRIVE guidelines recommended for reporting animal research, and Sex and Gender in Research

Laboratory animals	Male and Female mice at ~8 weeks of age were allocated equally into groups and sex was not considered in the study design.The R26-CAG-LSL-Sun1-sfGFP knock-in mouse on the C57BL/6J background was crossed with A2a-cre and Drd1-cre mice on a C57BL/6J background to generate Sun1-sfGFP;A2a-cre and Sun1-sfGFP;Drd1-cre mouse lines. All mice were purchased from Jackson Laboratory. Mice were housed on a 12-h light-dark cycle at constant temperature (23 °C) and humidity (40-60%) with access to food and water ad libitum.
Wild animals	No wild animals were used
Reporting on sex	Male and Female mice were used in this study. Sex was not considered because there was not suffcient n to make strong conclusions regarding sex as a variable.
Field-collected samples	The study did not include field collected samples
Ethics oversight	All animal procedures were conducted in accordance with the National Institutes of Health Guidelines as well as the Association for Assessment and Accreditation of Laboratory Animal Care. Ethical and experimental considerations were approved by the Institutional Animal Care and Use Committee of The University of Pennsylvania (Protocol # 805959). Male and Female mice at ~8 weeks of age were allocated equally into groups and sex was not considered in the study design.

Note that full information on the approval of the study protocol must also be provided in the manuscript.

ChIP-seq

Data deposition

☒ Confirm that both raw and final processed data have been deposited in a public database such as GEO.

☒ Confirm that you have deposited or provided access to graph files (e.g. BED files) for the called peaks.

Data access links<br>*May remain private before publication.*	All raw and processed sequencing data generated in this study have been deposited to NCBI Gene Expression Omnibus under accession number: GSE193673 (Gene Expression Omnibus under accession number: GSE193673 (https://www.ncbi.nlm.nih.gov/geo/query/acc.cgi?acc=GSE196673). The qPCR and cell counting data generated in this study are provided in the Supplementary Information/Source Data file. The N2a H3K4me3 ChIP-seq: GSE91043, (https://www.ncbi.nlm.nih.gov/geo/query/acc.cgi?acc=GSE91043); N2a H3K27me3 ChIP-seq: GSE107310 (https://www.ncbi.nlm.nih.gov/geo/query/acc.cgi?acc=GSE107310); NAc H3K4me3 and H3K27me3 ChIP-seq: GSE42811 (https://www.ncbi.nlm.nih.gov/geo/query/acc.cgi?acc=GSE42811), NAc RNA-seq: GSE121199 (https://www.ncbi.nlm.nih.gov/geo/query/acc.cgi?acc=GSE121199). Source data are provided with this paper.
Files in database submission	GSM5816009 N2a IgG 50K<br>GSM5816010 N2a IgG 500K<br>GSM5816011 N2a H3K4me3 50K<br>GSM5816012 N2a H3K4me3 500K<br>GSM5816013 N2a H3K27me3 50K<br>GSM5816014 N2a H3K27me3 500K<br>GSM5816015 D1 IgG control<br>GSM5816016 A2a H3K4me3 Epicyphr rep1<br>GSM5816017 A2a H3K4me3 Epicyphr rep2<br>GSM5816018 A2a IgG control<br>GSM5816019 D1 no antibody control<br>GSM5816020 D1 H3K4me3 Epicyphr<br>GSM5816021 D1 H3K4me3 Abcam rep1<br>GSM5816022 D1 H3K4me3 Abcam rep2<br>GSM5816023 D1 H3K4me3 Active Motif rep1<br>GSM5816024 D1 H3K4me3 Active Motif rep2<br>GSM5816025 A2a H3K4me3 Abcam rep1<br>GSM5816026 A2a H3K4me3 Abcam rep2<br>GSM5816027 A2a H3K4me3 Active Motif rep1<br>GSM5816028 A2a H3K4me3 Active Motif rep2<br>GSM5816029 A2a H3K27me3 Active Motif rep1<br>GSM5816030 A2a H3K27me3 Active Motif rep2<br>GSM5816031 A2a H3K27me3 Thermo Fisher rep1<br>GSM5816032 A2a H3K27me3 Thermo Fisher rep2<br>GSM5816033 D1 H3K27me3 Thermo Fisher rep1<br>GSM5816034 D1 H3K27me3 Thermo Fisher rep2<br>GSM5816035 D1 H3K27me3 Active Motif rep1<br>GSM5816036 D1 H3K27me3 Active Motif rep2
Genome browser session<br>(e.g. UCSC)	*Provide a link to an anonymized genome browser session for "Initial submission" and "Revised version" documents only, to enable peer review. Write "no longer applicable" for "Final submission" documents.*

Methodology

Replicates	2 replicates per cell type and antibody
Sequencing depth	~3-20 Million reads per sample Described above
Antibodies	CnR: anti-H3K4me3 (Antibody 1: 1:50 dilution, Abcam, Ab8580 (Figure 2); Antibody 2: 1:50 dilution, Active Motif, 39159 (Supplemental Figure 2); Antibody 3: 1:50 dilution, Epicyphr, 13-0041), anti-H3K27me3 (Antibody 1: 1:100 dilution, Active Motif, 39055; Antibody 2: 1:100 dilution, Thermo Fisher, MA5-1198) or IgG (1:100; Epicypher, 13-0042) CnR: anti-H3K4me3 (Antibody 1: 1:50 dilution, Abcam, Ab8580 (Figure 2); Antibody 2: 1:50 dilution, Active Motif, 39159 (Supplemental Figure 2); Antibody 3: 1:50 dilution, Epicyphr, 13-0041), anti-H3K27me3 (Antibody 1: 1:100 dilution, Active Motif, 39055; Antibody 2: 1:100 dilution, Thermo Fisher, MA5-1198) or IgG (1:100; Epicypher, 13-0042) peaks
Peak calling parameters	H3K4me3 peaks were called using MACS2 72 with parameters: -f BAM -g mm -B --keep-dup all -q 0.01. H3K27me3 peaks were called using SICER 73 with the command: sicer -t inbam -c control -s mm10. H3K27me3 peaks with false discovery rate less than 0.01 were selected. Overlapped peaks were counted using HOMER mergePeaks function 74 and visualized by Upset plots 75. Fraction of reads in peaks was calculated using a custom Python script.
Data quality	H3K27me3 peaks with false discovery rate less than 0.01 were selected.

Software	Bowtie2 v2.1.0, STAR v2.7.1a, cufflinks v2.2.0, deepTools v3.5.0, HOMER v4.11, MACS2 v2.2.7.1, Samtools v0.1.19, Rv4.2.2,, Picard v2.23.4, SICER v2.0 The custom scripts for the analysis can be found at https://github.com/HellerLAbeats/lCuRuS
